# Placement educators’ perspectives of international social work students’ sociopragmatic communication skills

**DOI:** 10.1007/s10459-022-10155-1

**Published:** 2022-08-18

**Authors:** Averil Grieve, Binh Ta, Bella Ross

**Affiliations:** 1 Student Academic Support Unit, Faculty of Medicine, Nursing and Health Sciences, Monash University, Caulfield, Australia; 2 Monash College, Melbourne, Australia

**Keywords:** International students, Sociolinguistics, Placement, Workplace communication, Work-integrated learning, Social work, Field education, Allied health

## Abstract

International students who speak English as an additional language report experiencing communication issues while completing their studies and work-integrated learning placements in a range of Anglophone countries, including Canada, the United Kingdom, the United States of America and Australia. To address this issue, accreditation and registration bodies for a number of health professions, such as social work and nursing, have advocated for increasing the test score requirements for university English language entry. However, from a sociolinguistic perspective, decisions concerning ways to address communication challenges need to take into account the unique communication skills required for functioning in specific workplace settings. It is therefore essential to identify the types of communication issues occurring during work-integrated learning opportunities (e.g. placement) and to then assess whether these should be addressed by raising general English proficiency or providing structured learning opportunities for profession-specific communication development within the course content. The present study uses sociolinguistic theory to examine placement educators’ perspectives on international students’ communication issues using the context of social work placement. It draws on the thematic analysis of interviews with 15 placement educators in Australia. One major finding is that international students’ general proficiency or ability to use specific linguistic tools (pragmalinguistic competence) is not a key area of concern for educators. The main challenge seems to involve the students’ understanding of sociocultural norms underlying workplace communication (sociopragmatic competence). This finding suggests that, rather than increasing English language entry requirements, universities need to provide international students opportunities to develop their sociopragmatic competence both before and during placement. The paper concludes with a set of recommendations aimed at supporting international students who speak English as an additional language to develop their workplace communication during their studies.

Demand for the internationalisation of higher education continues to grow, despite setbacks related to the Covid-19 pandemic (Institute of International Education [IIE], 2020). Key destination markets are Anglophone countries, such as the United States, the United Kingdom, Canada and Australia, which receive up to 47% of students who cross international borders to countries where they are not residents for the purpose of studying at a higher education institution (IIE, 2020, Tight 2022). However, a number of these international students report experiencing communication issues, especially while completing health professions work-integrated learning (WIL) placements in Anglophone countries (e.g. Law et al., 2022; Eden et al., 2021). To address this issue, accreditation and registration bodies for a number of health professions have advocated for increasing university English language entry test score requirements (e.g. AASW, 2020). However, from a sociolinguistic perspective, decisions concerning ways to address communication challenges need to take into account the specific communication skills required for functioning in specific workplace settings (Benzie, 2010).

The acquisition of professional communication skills specific to workplace settings is part of a large set of student graduate employability competencies (Jackson, 2014). In the health professions, compulsory work-based placements play a central role in ensuring students graduate with these key attributes (Cord & Clement, 2010; Gamble et al., 2010). However, in terms of professional communication development, access to placement seems to be a double-edged sword. On the one hand, it is during placement that students can develop key workplace communication and employability skills (Jackson, 2014). On the other hand, WIL educators and students indicate that communication issues can inhibit learning and teaching during industry-based learning opportunities (Ross et al., 2019; Ta et al., 2020).

Using the context of social work placement, this study examines placement educators’ perspectives on the types of communication challenges faced by international students. The term ‘international student’ refers to all individuals holding a study visa or equivalent (e.g. a Subclass 500 in Australia, a student visa F in the United States of America or a study permit in Canada) while enrolled in a full-time tertiary education course (Department of Home Affairs, n.d.; Government of Canada, n.d.; US Department of State, n.d.). The term ‘placement’ (also known as ‘fieldwork’ or ‘field education’) is used to denote any WIL experience that aims to integrate theory with practice and is a prerequisite for obtaining a health professions degree (Patrick et al., 2008). ‘Placement educators’ are individuals trained in a specific health profession who are responsible for the supervision of students’ learning during work placement. They do not necessarily have any training in education or language acquisition. For example, in the context of social work in Australia, placement educators are qualified social workers who have a minimum of two years full-time practice experience, and are eligible for full AASW membership (AASW, 2020).

As discussed in detail below, the role of placement (in particular) or WIL (in general) on the development of communication skills, has been researched from the perspectives of both students (e.g. Blackmore et al., 2014; Gursansky & Le Sueur, 2012) and educators (e.g. Jackson 2017; Ross et al., 2019; Ta et al., 2020; Grieve et al., 2021). However, while these studies all indicate that communication skills development is both a key issue and outcome of placement experience, there is a paucity of research that identifies the specific social and linguistic communication issues that occur and need to be addressed during placement. Filling this research gap, this study uses sociolinguistics to classify communication issues identified by placement educators. It offers insights that have significant relevance for determining how to best support international students to develop employability and workplace communication skills, including decisions as to raising English language university entry requirements.

## Sociolinguistic theory and communicative competence

From a sociolinguistic perspective, communicative competence encompasses the knowledge and skills that are required for communicating appropriately and effectively in different social contexts (Hymes, 1972). The two components of communicative competence are pragmalinguistic competence and sociopragmatic competence (Leech, 1983). Pragmalinguistic competence refers to the knowledge and ability to use linguistic tools (e.g. grammar and vocabulary) and pragmatic strategies to convey meaning (Haugh & Chang, 2015). Sociopragmatic competence requires speakers to understand sociocultural norms underlying the communicative events they participate in and apply their pragmalinguistic knowledge to achieve their communicative goals (Haugh & Chang, 2015).

Research in the field of second language learning has provided consistent evidence that it takes a long and highly variable time for second language speakers to develop communicative competence as it concurrently entails acquisition of unfamiliar sociocultural norms (Ellis, 1992; Hill, 1997; Trosborg, 1995). Developing communicative competence involves providing learners with authentic contextualised language input (e.g. placement interactions) and explicit teaching of sociocultural norms related to specific communication contexts (Ishihara, 2010; Ishihara & Cohen, 2010; Kasper & Rose, 2001; Rose, 2005; van Compernolle & Williams, 2012). The role of authentic contexts on international students’ communicative competence is important; the longer the duration of an international student’s language immersion and the wider the range of contexts a student is exposed to, the wider sociopragmatic variations they develop (Devlin, 2019). This finding supports the view that developing communicative competence requires intensive exposure to authentic contexts.

Sociopragmatic knowledge is closely related to professional and workplace cultural practices; it is socially-specialised use of language that is goal-directed and evaluative (Leech, 1983, p. 11). For example, speakers can intentionally use their sociopragmatic skills to express the extent to which they belong to a profession or workplace. Concurrently, their sociopragmatic language skills are evaluated by listeners in order to assess the extent to which the speaker is a valid member of the profession. Sociolinguistics, therefore, offers a useful lens to view workplace communicative competence, as it can help identify whether pragmalinguistic or sociopragmatic aspects of communication are the most challenging for international students. By ascertaining this, evidence-based decisions concerning ways to address the issues faced by students can be made. If the communication challenges are essentially pragmalinguistic, raising the English language course entry requirements may assist students to have a firm understanding and application of the tools of language (e.g. vocabulary, syntax) before they begin their studies. Comparatively, if the issues are predominantly found to be sociopragmatic and linked to highly specific communicative situations, a key means of addressing the issues is to increase the opportunities students have for authentic workplace communication experiences and provide explicit teaching of sociocultural norms within those professions.

## English language entry tests

English language entry tests, such as the International English Language Testing System (IELTS) Academic test, are designed to assess international tertiary-level students’ abilities for future university study (Benzie, 2010; Huang, 2021). These tests provide a point-in-time snapshot of the test taker’s general English proficiency for universities wanting to predict a student’s ability to commence and complete studies in an English-speaking educational environment (Hamid, Hoang & Kirkpatrick, 2019).

English language entry tests do not aim to assess a student’s pragmalinguistic or sociopragmatic abilities (Roever, 2011). Instead, they focus on assessing a student’s general academic language proficiency (Douglas, 2000) and ability to use the language for functional communication in academic contexts where English is the language of instruction (Dashti & Razmjoo, 2020). The tests are, therefore, not necessarily good indicators of a student’s ability to operate within a specific discipline (Schmitt, 2005) or workplace.

Despite none of the tasks of English language entry tests closely corresponding with what an employee would be expected to do in many jobs (Pearson, 2019), they are increasingly used by employers to both predict and assess a graduate student’s ability to function in the workplace. This includes social work in Australia, whereby for both entry to an accredited Master of Social Work course as well as graduate membership to Australian Association of Social Work, international students must have a minimum score of 7.0 or higher in each component (listening, reading, writing and speaking) of the Academic IELTS test (AASW, 2020). Similarly, to practise as a registered health or care professional in the United Kingdom, an international student graduate must achieve an overall IELTS of 7.0 (with no band below 6.5) or its TOEFL equivalent (Health and Care Professions Council, n.d.). However, both the predictive validity and the construct validity of the use of English language tests for professional purposes has been questioned (O’Loughlin, 2008), with the possibility of a disjunct between the language proficiency assessed by the test and the pragmalinguistic and sociopragmatic skills required to be a graduate professional.

## Communication skills development on placement

Placement experience is beneficial to international students for developing professional networks (Gribble & McRae, 2015; Ng et al., 2019), and their ability to communicate with people with diverse cultural backgrounds (Kelly, 2017; Tran & Soejatminah, 2016). This suggests that workplace communication competence is not a fixed attribute, it is rather an outcome of placement experience.

Despite being considered a dominant aspect impacting student placement success (Jackson, 2014), communication skills have been, at best, only poorly defined and understood. Jackson & Chapman (2012), for example, define only the situations in which oral communication skills are to be displayed (ability to give and receive feedback, speak publicly, participate in meetings and verbally communicate with others in an effective manner). However, this definition only focuses on broad communicative events, without any specific reference to the components of oral communication skills that are to be displayed, nor to a reliable means of assessing effectiveness. More importantly, definitions provide little or no reference to the sociopragmatic skills a student typically needs to draw on in order to calibrate their language according to nuanced changes in the communicative event. For example, a student’s ability to give and receive feedback effectively is dependent on the place in which the conversation takes place, the power differentials between the interlocutors, how familiar the interlocutors are with each other, the content of the feedback being received or provided, and the end goal of the feedback session (Brown & Levinson, 1987; Hymes, 1972) - all of which are socioculturally determined by the interlocutors themselves and by the broader culture of the environment in which the interaction takes place. When communication competence is poorly defined, there is a risk of underestimating the complexity of the student’s language use and, in turn, misjudging international students’ challenges with communication in workplace settings. The final two paragraphs of this section review the growing body of research on the communication challenges faced by international students on clinical placement.

A number of communication challenges experienced by international students on placement, especially clinical placement, have been identified (Deegan & Simkin, 2010; Nash, 2011; Patrick et al., 2008; Rai, 2002; Spooner-Lane et al., 2011). These challenges include understanding local colloquialisms, accents, idioms, abbreviations and discipline-specific language (Nash, 2011). International students’ communication difficulties may also be linked to cultural differences in workplace interaction (Harrison & Ip, 2013; Rai, 2002; Taylor et al., 2000; Zunz & Oil, 2009). For example, international students are reported to face issues of cultural adjustment in terms of both social conventions (e.g. how to address placement educators or conduct small talk, acceptable topics of conversation and whether it is appropriate to criticise a supervisor) and culturally-specific beliefs and values (e.g. a clash of values regarding what is acceptable in the host country vs. the home country) (Harrison & Ip, 2013).

Research drawing on placement educators’ perspectives often depicts international students’ communication skills as being deficient (cf. Harrison & Ip, 2013) and working with international students is seen by educators as challenging and time-consuming (Ta et al., 2020; Ross et al., 2019). Challenges reported by placement educators include international students’ weak communication skills (Jackson, 2017; Mantzourani et al., 2015) and a lack of understanding of workplace culture (Fotheringham et al., 2018; Jackson, 2017; Mantzourani et al., 2015). This negative framing suggests an expectation that international students possess adequate communication competence prior to placement. While this expectation seems fair in relation to general language proficiency, expecting international students to immediately be able to communicate effectively in a workplace environment is not always realistic. As outlined in the previous section, it is through placement that workplace communication competence is developed, and this is applicable to all students, not only those classified as international or culturally and linguistically diverse students (Cord & Clement, 2010; Jackson 2014; Martin, 2014).

## Research aims

Using a sociolinguistic lens, the present study explores the types of communication issues that placement educators encounter when supervising international students from culturally and linguistically diverse backgrounds. It is based on an understanding that authentic contexts are crucial for developing communicative competence and teaching employability skills (UKCES, 2008, p. 62). As placement provides authentic work contexts, it could be a conducive environment for developing employability and workplace communicative competence, particularly if learning contexts are constructed to optimise sociopragmatic skills development.

## Research methods

This qualitative research takes an interpretivist ontological perspective, whereby realities are seen to be socially constructed and, therefore, both subject to and malleable to change. In all cases, the information and findings provided in this paper reflect the diverse realities of the participants and researchers, which are deeply embedded in a range of individual and cultural contexts.

This article reports on a section of data from a larger research project, which used survey and interview data to examine social work placement educators’ experiences and professional development needs for supervising international social work students. Ross et al., (2019) reports on the findings of a pilot survey informing a nationwide survey, Ross et al. (2020) reports on Section (ii) and Grieve et al. (2021) reports on Section (iii) of the nationwide survey and interview data that pertained to specialised training for the supervision of international students. Ta et al. (2020) reports on interview responses that focus on placement educators positioning of themselves as educators. Comparatively, this paper reports on interview responses to questions pertaining to placement educators’ perceptions of student communication challenges.

The 15 semi-structured one-on-one telephone interviews between placement educators and the second author were used as the basis of analysis for this paper. The interviews ranged between 18 and 65 min in duration with questions focussing on the placement educator’s experiences of supervising international students. There were 13 female and two male educators.The mean years of supervising was 2.9 and the mean number of students supervised was 8.5. Educators had experience supervising between 1 and more than 15 international students (mainly from China) and had supervised social work students from between 2 to over 30 years. While two placement educators were concurrently educators at a tertiary institution, all others were employed directly by the agency providing the placement opportunity. The interviews were audio-recorded and transcribed verbatim by a paid transcription service. Ethics approval for the research was granted by the Monash University Human Research Ethics Committee (Project No. 0254).

After transcription, all comments focusing on the students’ communication were identified (n = 116) and coded for the type of communication to which the placement educator was referring. While explicit reference to international students’ sociopragmatic and pragmalinguistic skills was not made, these themes emerged when the placement educators recounted the communication challenges experienced by their international students, and how they supported international students to overcome these challenges.

The coding system was informed by Leech’s (1983) framework for studying language as a communication system and integrating knowledge of formal language systems (e.g. grammar, vocabulary) with knowledge of using language to have meanings in communicative situations (e.g. language in use).

**Fig. 1:**
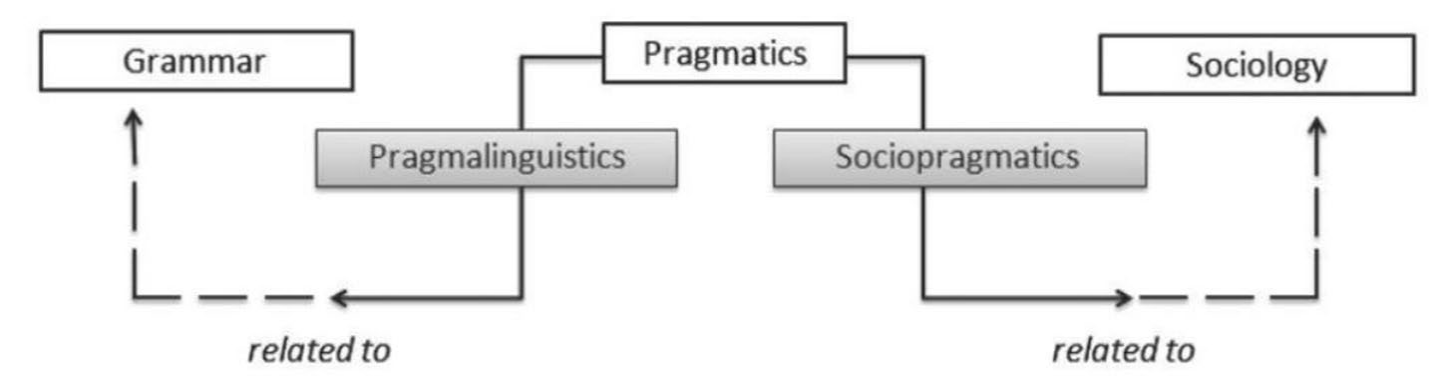
Leech’s (1983) distinction between pragmalinguistic and sociopragmatic knowledge (Souce: Timpe-Laughlin, Wain and Schmidgall, 2015)

As per Leech’s (1983) framework (Fig. 1), comments made by the placement educators concerning the students’ communicative abilities were initially coded as being either of a pragmalinguistic or a sociopragmatic nature. Pragmalinguistic comments were those that were related to the students’ knowledge of formal aspects of language. These included general syntax, semantics, phonology and vocabulary. Interview comments that were coded as being sociopragmatic focused on the ways in which the pragmalinguistic tools of language were employed in situations that were specific to the social rules of language use in the profession or agency (e.g. small talk topics, taboos). A third code (‘unspecified’) was developed for those comments in which it was not clear whether the comment related to sociopragmatic or pragmalinguistic competence.

As shown in Table 1, the three first level codes were further coded into six possible second level subcodes, focusing on a communicative skill area. Second level codes could then be further coded into specific descriptors on two more levels, resulting in a four-tiered coding system for all comments relating to language.

**Table 1:** Coding system

Level 1	Level 2	Level 3	Level 4
sociopragmatic pragmalinguistic unspecified	speaking writing listening reading body language unspecified	academic skills detail genre gist grammar orthography pronunciation register roles unspecified vocabulary	age argument structure capitalisation case notes clarity disability elderly eye contact feedback feelings formal/informal gender idioms initiative instructions medical notes meeting minutes meetings numbers physical distance referencing reports seeking help/clarification sentence structure showing empathy slang spelling tense terminology topic appropriacy unspecified willingness to speak workplace expectations

## Intercoder reliability

Inter-coder reliability was conducted, whereby the first, second and third authors blindly coded a random subset of the data (34.5%) and then compared and discussed any discrepancies. The full data set was coded by the first author and then two rounds of intercoder reliability with the second and third author were conducted in which the second and third authors each coded 20 quotes. Discrepancies were discussed and subsequent changes were made to the coding system. After two rounds of inter-coder reliability (Table 2), agreement of over 84% was achieved for all four levels of coding and 100% agreement could be reached after discussion. This was considered to be an “excellent agreement beyond chance” (Banerjee et al., 1999, p.6) and the coding system was, therefore, considered to be reliable.

**Table 2:** Intercoder reliability

	Pre-/Post-	Level 1 n = 20	Level 2 n = 20	Level 3 n = 20	Level 4 n = 20
Round 1	pre-discussion	15 (75%)	14 (70%)	14 (70%)	11 (55%)
	post-discussion	20 (100%)	20 (100%)	20 (100%)	20 (100%)
Round 2	pre-discussion	17 (85%)	18 (90%)	16 (84%)	16 (84%)
	post-discussion	20 (100%)	20 (100%)	20 (100%)	20 (100%)

## Findings

**Fig. 2:**
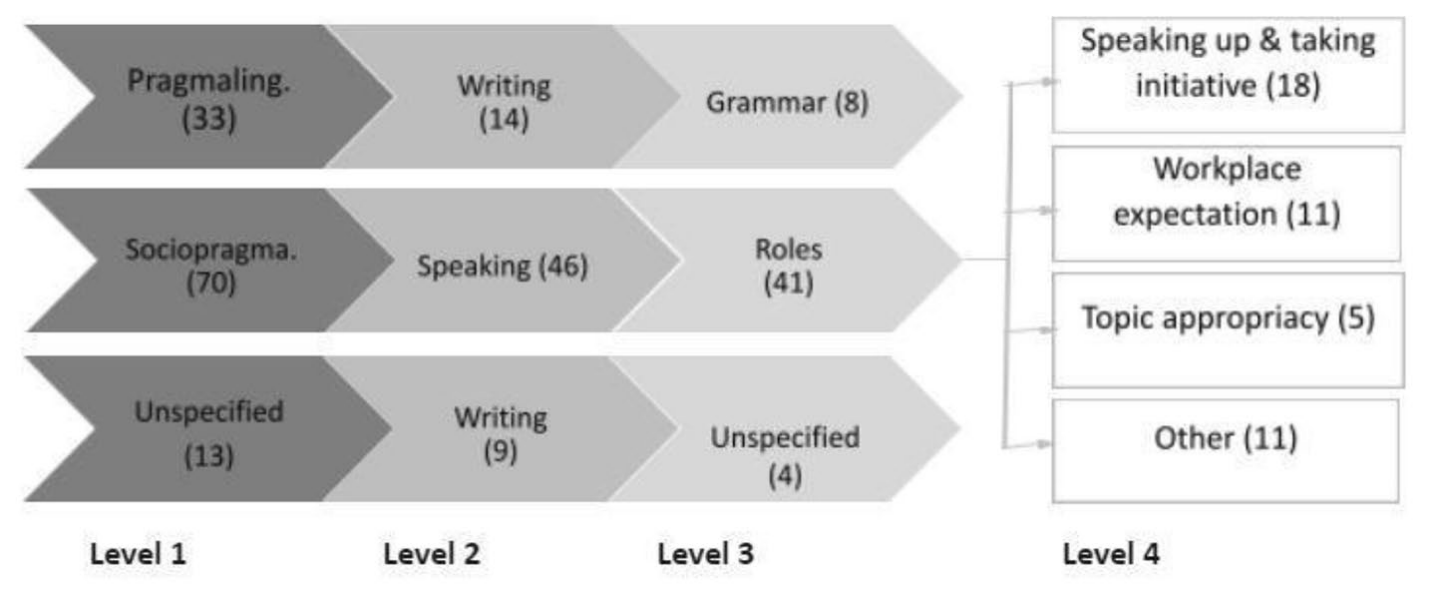
Coding level results

As shown in Fig. 2, of the 116 comments coded, the majority (61%) focused on the sociopragmatic skills of students supervised by the placement providers and only 28% were identified as being related to pragmalinguistic knowledge and ability to use linguistic tools (e.g. grammar and vocabulary). The pragmalinguistic issues perceived as most prevalent by placement educators were written discourse (42%), with a specific focus on grammatical competencies (54%). For the most part, educators were not able to express what the grammatical issue was, but emphasised that grammatical mistakes were impeding clear expression of meaning:

And the grammar’s very bad. Often [...] I don’t even understand what they’re trying to say and that’s pretty common with most of our students from international. Some of them are great but the majority are really struggling with their written (Int9)

Of those classified as sociopragmatic competence issues, 66% focused on spoken interaction, which was concurrently the most prevalent Level 2 code (49%) across all Level 1 codes. The coding indicated that issues identified as being associated with spoken sociopragmatic abilities typically related to the roles of individuals in the agency (Level 3). The theme of ‘roles’ was then further specified in Level 4 codes as referring to student’s willingness to speak, ability to take initiative, understanding of workplace expectations, and knowledge of which topics are most appropriate for the setting (Level 4). Based on these overall findings, the placement educators clearly indicated a specific area of interaction that was of the greatest importance to their experiences and abilities to supervise international students, namely the students’ sociopragmatic understanding of work-based roles. This paper will now provide an in-depth discussion of the ways in which placement educators further defined this understanding of roles and how this impacts on students’ abilities to communicate during placement.

## Speaking up and taking initiative

Speaking up and taking initiative are the most common student communication issues identified by the placement educators. They claim that, compared to local students, international students are more likely to be softly-spoken, shy and underconfident and not likely to ask questions or speak up about the issues they experience (8 quotes). They also indicate that international students struggle with initiating projects and taking actions when problems arise (10 quotes).

I think a lot of international students really struggled with initiating projects and initiating ideas, initiating interventions. (Int10)

[They’re struggling with] you know, when to speak up, and when not to speak up, when to show initiative. And they struggle with that. (Int15)

These two students again – were both very softly spoken and their assessments were excellent and their communication was always respectful. There were no concerns regarding their communication with patients or family, but it was again about, I think, more confidence when working with the interdisciplinary team. (Int3)

While Int10’s quote indicates that the educator believes that international students struggle with initiating ideas, Int15’s and Int3’s quotes suggest that not knowing when it is appropriate to show initiative is the main issue. The issues surface predominantly when the student is interacting with colleagues who are above them in the workplace hierarchy. This indicates that international students might have the ability to take initiative, but it is their uncertainty about sociocultural appropriateness within workplace hierarchies that prevents them from speaking up.

When accounting for the issue of international students “not speaking up” or “not showing initiative”, the placement educators suggest various factors, including not knowing workplace expectations, not feeling confident, and being afraid of making mistakes. For example, Int10 considers workplace awareness, fear of making mistakes, and fear of disrespecting workplace hierarchies as three possible factors.

I’m not sure whether it’s because, you know, they didn’t necessarily have a great awareness of the agencies, and lack understanding about the rules and regulations around it [...] or whether it’s that they’re afraid of doing the wrong thing and there being big consequences for that. [...] And I felt like there were a lot of students terrified that they were going to do the wrong thing, muck something up, step outside their station I guess, outside their role. (Int10)

Int6 indicates that the students’ low levels of sociopragmatic knowledge in terms of what is ‘proper’ for specific workplace situations can be misinterpreted and result in less learning opportunities being offered to international students. As shown in Int6’s quote below, although social work students in a school setting had ideas, they did not take their ideas to the school principal, which was interpreted as a sign of lacking confidence. Based on this interpretation, the principal did not challenge the students, the consequences of which were missed learning opportunities.

In the last school the students did not show very much initiative with the principal. [...] they wanted to do things but when I asked them, you know, had they taken it to the principal, had they gone in and asked for permission, they didn’t ask the question and they didn’t show the initiative because they felt like it wasn’t proper. So they just waited until somebody asked them and the thing is the principal interpreted that as they didn’t have the confidence to do it, so he didn’t push them. (Int6)

The issue of students not speaking up is claimed to pose a challenge for the educators to provide support as they do not understand what is happening and do not have time to follow it up. This is illustrated in the following quote:

For me, that makes it really difficult because I can’t chase a student all the time and check every five minutes, are you okay? Do you understand? What do you - you know, that sort of thing. I need somebody to be more proactive. (Int12)

Similarly, as shown in the quote below, when students do not speak up, educators find it challenging to assess the student’s knowledge and skills,:

They are often required to participate in group discussions. So often they’re very shy and they find it very hard to speak up. Like, so I have to make it really clear that I’m assessing their performance based on their verbal participation. And then if they’re shy and they don’t participate, then I can’t assess what they know and then I might be required to ask them to do more work requirements so that they can show me what they know. Because I need to hear it, I need evidence but if I can’t see the evidence. (Int6)

The above quotes reveal that the issue of speaking up among international students is a practical problem that placement educators need to address. The placement educators indicate that a key means of addressing sociocultural misalignment is having a direct conversation about sociopragmatic norms with the students. For example, by discussing the above-mentioned student-principal interaction directly with the students, the placement educator (Int6) came to understand the underlying situational and sociopragmatic reasons for the students’ behaviours. The student’s actions were seen as cultural differences in workplace deference and not a reflection of English language proficiency.

I don’t think that’s a verbal skill issue, I think that it is cultural. Because I think when I talked to the students afterwards, they said “Oh no, no we didn’t think it was respectful to ask the principal.“ Whereas in Australia, the principal was seeing that not asking as a lack of initiative. (Int6)

This quote suggests that there was a risk of believing that the issue was linked to verbal language skills. However, after enquiry with students, the educator realised it was a misalignment of sociocultural understanding (i.e. sociopragmatic competence). Other educators independently tell similar stories concerning reflective discussions they have with students.

And the students have said, “Well, in our culture, we don’t do something unless we’re asked to do it.” So it’s about understanding the different cultures, being prepared for that. (Int15)

The two students reflected on this – they were separate but they reflected similarly that the cultural backgrounds that they had, you were more deferential to a clinical staff and to other people. (Int3)

Another common method to address communication issues is for the placement educator to explain their expectations about the students’ speaking performance. For example, using direct reported speech, the following educator reports what she said to the students, whereby she uses the construction ’you need to’ to communicate her expectations to the student.

And, it has taken a lot of work to try and break down the barriers, and say, “It’s fine, you need to let us know what is happening, and what you’re thinking and feeling. Because, if you can’t tell us why you can’t do these things, then how are we supposed to help you as [placement educators]? (Int13)

Similarly, the following quote indicates that Int12 uses ‘you need to’ and an imperative construction with ‘write’ to address sociocultural issues.

I asked, you know, you need to ask curious questions. You need to do this. Write yourself a script [...] We did a number of supervision sessions around that, but it didn’t change. (Int12)

Interestingly, the above quote also indicates that the one-way approach of communicating expectations from the supervisor to the student does not work. Asking the students to change their speaking behaviours without addressing the sociocultural matters underlying the behaviours will not necessarily result in fruitful outcomes. Comparatively, as shown in the above quotes (Int3, Int6 and Int15), engaging in open discussions with international students may work best because these interactions allow students’ views to be explored and cultural differences to be addressed.

The findings suggest that sociocultural understandings of workplace hierarchies have an impact on language use. These quotes also suggest that engaging in two-way exploratory conversations with international students to understand their own accounts of their communication behaviours can enrich both the students’ and the educators’ perspectives.

## Workplace expectations

Closely linked to issues of deference and speaking up are student communication challenges precipitated by a mismatching of workplace expectations, many of which are particular to the culture of the agency setting or are assumed knowledge for professional behaviour in Australian workplaces. Three key sociopragmatic issues indicated by educators are: (1) knowing when to provide information or explanations; (2) knowing who to contact or speak to about certain work-related topics; (3) recognising the type of communication required; and (4) clearly distinguishing between expressing collegiality and friendliness in communication.

The placement educators indicate that international students may not be fully aware of highly situation-specific and multifarious sociopragmatic practices that guide individuals to know what, to whom and how to communicate in professional settings. In the quote below, Int6 clearly explicates the layers of sociopragmatic knowledge necessary for communicating absences. These include knowing whether it is necessary to engage in a communicative event (informing the workplace of an absence due to illness), what is considered an appropriate medium (sending a message or ringing), and who is considered to be the most appropriate recipient of the information (the educator or the workplace).

I think they need to understand employability skills and the employment culture in Australia. So, you know, when you ring [00:26:08] you ring up and tell people that you speak and all that sort of information and how you conduct yourself. So sometimes students don’t ring the workplace and say they’re sick, they send a message to me and then they don’t say anything to the school. And I make it really clear that their first priority is to tell the workplace, not me. (Int6)

Another educator (Int7) also indicates that some international students do not communicate information well simply because they do not know how to do it appropriately (“Who to report, yeah, how to report it, yeah.”), which results in confusion for both the educator and the students (“They get, I get, confused, they don’t know if they have to.”).

Even if the above-mentioned steps of knowing what to say to whom via which medium are fulfilled according to the specific workplace expectations of the placement agency, the student will still need the necessary sociopragmatic skills to understand how to formulate their message appropriately and negotiate the terms of the absence:

They just say, “I need to take a week off” and they go and they just think that it’s – they don’t understand that you need to explain. That isn’t something that you just decide, you have to actually get permission from the university and you have to make that time up and all those sorts of things. (Int6)

Int15 indicates that a possible explanation for confusion surrounding workplace communication is that the students may be exposed to “mixed messages” about friendliness and collegiality in the workplace, which may, in turn, impact on the sociopragmatic choices the student makes in English. Int15 indicates that in making such choices there is a nuanced differentiation in communicating an overall collegial friendliness and being friends.

And they think they’re friends as opposed to friendly. It’s a different culture, you know, being friendly. And I think they kind of don’t quite understand the nuances there. So they don’t pick up on the subtleties of - they actually don’t pick up on the subtleties of - what’s it called - of the workplace culture. I think they also kind of get confused with that means that you can come to work, it’s all right if you’re late. I think they get really mixed messages about that. (Int15)

Such nuanced language use can have high-stakes repercussions for a student. For example, Int6 indicates that they “had a student who nearly failed her placement […] because she did not understand how to communicate with her supervisor verbally about what was going on and she didn’t understand what the expectations were around the behaviour.”

In order to overcome such difficulties, placement educators indicate they use review meetings to talk about the issues with the student individually (Int11) or as an unspecified group (Int6). The educators claim that they explain the issues to the students and also set clear rules and expectations, however, as indicated in the quotes below these methods of explanation are not always successful.

Look, you know, I made him come in early at times, I made him stay back late. We talked a lot about, “This is your commitment to this course, this is your commitment to this organisation,” but if you were to say to me, “Would I have him back?” absolutely no I wouldn’t, based on that. (Int11)

Even though it was explained to her, she just didn’t really [...] she really didn’t have the verbal skills to understand how to talk to people and explain and understand what people expected of her in the workplace. (Int6)

## Topic appropriacy

Topic choice that is specific to the culture of the profession and agency is also identified by placement educators as a sociopragmatic hurdle for students. This related both to interactions with colleagues as well as communicating with clients.

Linking directly to the mixed messages of friendliness versus collegiality, international students are identified as possibly following different sociopragmatic rules of informal interactions in the workplace. For example, Int15 identifies engaging in small talk to develop rapport and hedge problem-focused conversations as being a point of misunderstanding between students, colleagues and placement educators. According to Int15, the choice to engage in small talk is, for some students, also linked directly to culturally-specific hierarchical positions in the workplace.

They don’t believe in this small chat, and wining and dining, and establishing a kind of rapport. For them, you can go straight into, “Right, what’s the problem? What are we here to talk about?” So I think they struggle with understanding that, for us, it’s kind of the reverse. If you want to get somewhere, if you want to achieve, if you want to build good relationships, you have to invest in getting to know someone. Whereas, a few Chinese students have said to me, “Oh, no, [name removed], that’s not how it’s done. You sit down. You acknowledge each other. You acknowledge that you’re at different levels, if that’s the case. And you can get on with what you need to do.” (Int15)

Similar to the data focusing on provision of information, even if a student does engage in small talk, issues can still arise in terms of what to say and how to talk about specific topics. As shown by Int1, this can be based on local knowledge of significant cultural practices (e.g. NRL and AFL football leagues, local music) to which students have not had extensive exposure, and cannot, therefore engage in rapport building conversations with their colleagues.

When people talked about the football. People talk about local band, or the music that they enjoy. So then when you build connection or build relationship with the people we work with, so they may ask you “which footy team do you support?” And as an international student, sometimes you just come – you’ve only been here for six months – “I’ve got no idea what you’re talking about, footy league teams”. So you know – even to try to understand the NRL, AFL – this culture is very typical day-to-day culture, just that you are someone who grew up in another country and you’re not familiar with some local [...] pop culture. (Int1)

Issues also seem to arise when the international student initiates topics in casual workplace conversations that “throw staff off guard” because they are not expected in the local workplace setting. These may include questions that are considered too personal in the workplace culture of the agency, but are considered appropriate in the students’ previous professional interactions.

There were many times when he would ask questions that perhaps weren’t culturally appropriate. I think staff were sort of thrown off guard and they – we talked about it in supervision and it wasn’t that he – I think he – he couldn’t work out what was appropriate and what wasn’t [...] he might ask quite personal questions of a staff member. You know, are you married? Have you got children? But it’s not the sort of thing that we – that is asked when you get in the car with someone when you go on a visit. (Int11)

As indicated above by Int11, supervision meetings are used as a means of talking about these issues, as is monitoring of the student in situations where they may ask questions that are considered inappropriate (“just had to watch the questions”). Int10 also advocates for directly explaining to students the need to, at least, show an interest in local small talk topics, so that they can have relaxed conversations on placement with local staff, e.g. finding a football team:

I’ve always said to students, “One of the first things you need to do is find a football team that you’re going to say that you support, or a team that you follow. You don’t have to like football.” You don’t have to know anything more about it other than there is this one team and that’s my team. And that actually breaks ice. It makes people relax. (Int10)

## Other issues

There are a range of issues that are only mentioned once or twice or are not specified in detail. One key issue, that will be mentioned in this paper due to its central role in social work placement, is students’ sociopragmatic understandings of ways to adjust their language to interact with different clients, particularly the elderly or clients with disabilities. For example, Int4 indicates that students do not adjust the complexity of their vocabulary while at the same time remaining professional.

One of the things that we’ve found is because we have disability and age [...] So learning to adjust their level of communication to simple word usage, to a professional level, so you have a very broad range in there. Because if you have somebody with an intellectual disability, on a professional level, you can’t use certain words that they’re not going to be able to understand. So you have to break it down into more simple English verbal forms of communication. (Int4)

But in those situations, because we work with people with disabilities, sometimes they need extra support. We need someone who will be able to communicate or use different words to explain the bill to the person. (Int1)

The complexity of this issue is highlighted by Int13 who indicates that different sociocultural norms of communication need to be combined with intersectional identities. In this case, the student needs to negotiate the intersectional identities of age and gender with socioculturally specific norms of deference (e.g. respecting elderly people) and topic choice (e.g. talking about feelings). According to the educator, the student was uncomfortable talking about emotions to elderly men because she was brought up in a culture where young women are not supposed to question men about how they are feeling and managing things. As a result of this, she did not successfully complete her placement.

A big issue was that she was Chinese, and she was saying to me, “In the Chinese culture you respect your elders. You don’t question.” She was a female. She was brought up not to question men, with what men have to say, and that in her culture how she was raised, you don’t really talk about your feelings, and how you’re coping with things. [...] She was uncomfortable talking to men and to older people, because you respect those people, you don’t ask them questions about how they’re managing things at home, and how they’re feeling being in hospital. (Int 13)

The sociocultural hurdle of communicating emotions while working with clients is identified as an issue regardless of the client type. For example both Int13 and Int10 indicate their students found it difficult to work with, discuss, and address the clients’ feelings.

With my particular student, she didn’t feel comfortable providing support to someone because the way she was raised, you don’t talk about your feelings. So, to try and help someone else to manage their feelings was just something that she wasn’t able to do. (Int13)

Similarly, Int10 indicates that students struggle to engage in in-depth expressions of their own emotional reactions to events on placement. This is not due to their general proficiency in English, but due to the sociopragmatic rules of integration concerning particular topic areas in a professional work environment.

It’s very hard to get those nuanced discussions from the international students. If we said to them, “Well how did you feel about that?” And social work, unlike accounting or something, we do want to know how did you feel, what did you think, how did you feel, because that’s how you reflect on your practice and your biases and everything. (Int10)

## Discussion and conclusions

The findings of this research indicate that placement educators predominantly identify communication issues of international students on placement as being sociopragmatic in nature. More specifically, they perceive sociocultural and sociolinguistic understanding of professional roles and workplace expectations as the key factor impacting on the student’s language use, particularly in terms of their spoken discourse. Such understanding is complex and multi-layered in that it entails not only knowledge of what to say to whom, when and via which medium, but how to choose specific tools of language to formulate the message appropriately. A student’s lack of role or workplace clarity can lead to hesitancy to initiate or be involved in workplace interactions. This can create a perception of international students lacking confidence or initiative, asking rude or inappropriate questions or failing to engage in conversations that are expected of them in the placement setting. In turn, this may lead to reduced learning opportunities and placement educators not feeling able to provide support or assess students according to their knowledge and skills in the profession. The findings also indicate that assimilationist approaches of explaining issues to students and setting clear rules and expectations about workplace norms of interaction are less effective than approaches that engage in two-way exploratory conversations about sociocultural and sociolinguistic norms underlying particular student behaviours.

These findings clearly indicate that placement provides international students with highly authentic and contextualised professional language input (UKCES, 2008) and placement educators with the opportunity to explicitly teach sociocultural norms (Ishihara, 2010; Ishihara & Cohen, 2010; Kasper & Rose, 2001; Rose, 2005; van Compernolle & Williams, 2012). While the cultural and communication challenges identified by the educators align with those identified in previous research (Harrison & Ip, 2013; Nash, 2011; Rai, 2002; Taylor et al., 2000; Zunz & Oil, 2009), the educators in this study do not all adopt a deficit framing. Instead the educators seem capable of clearly identifying communication issues as being mostly linked to either sociocultural knowledge (sociopragmatic) or grammatical competence (pragmalinguistic). It is, however, important to note that the identification of these issues is based on the educator’s own assumptions and expectations of communication norms, which themselves are deeply embedded in the educator’s individual perception of the social world (i.e. *habitus*). Similarly, the educator’s self-assumed role in both setting and teaching these communication competencies to students resides in an implicit culturally-defined power differential between teacher and learner (Farias et al., 2021).

This paper also indicates that the key issue does not seem to be in the identification of communication challenges, but in approaches to address these issues so that the rich opportunities for developing workplace communication during placement (Cord & Clement, 2010; Jackson 2014; Martin, 2014) are fully mobilised. While some teaching approaches for this development are discussed by the educators, they seem to be implemented in a somewhat haphazard fashion and with varying success. This raises issues of equity for international students, whereby the learning opportunities they can access and their ability to successfully complete placement is reliant on the placement educator’s ability to not only recognise, but also address sociolinguistic issues so that they are harnessed as learning and development opportunities.

Those educators who are most effective in assisting students to gain sociopragmatic confidence focus their teaching on gaining mutual understanding rather than a one-sided explanation of norms of interactional behaviours. Unsuccessful one-sided directives with little or no mutual engagement may simply perpetuate misunderstandings and increase both student and educator frustrations during placement. Despite many of the educators being capable of identifying pragmalinguistic and sociopragmatic language issues, placement educators are, first and foremost, experienced professional social workers, in many cases with no requirement for having a background in education or language acquisition (AASW, 2020). This indicates a need for either university-driven specialised training of placement educators working with international students (Grieve et al., 2021; Ross et al., 2020; Ta et al., 2020) or support for international students by discipline-specific specialists in language learning and teaching (Arkoudis et al., 2012). Specialised training should assist educators to move their teaching away from assimilationist approaches (Ross et al., 2019; Tran & Soejatminah, 2016) and gravitate towards reciprocal learning of sociocultural norms of interaction and mutual formation of new cultures and realities (Grieve et al., 2021). Key to the success of such training is reflective practice that highlights the educator’s ‘hidden’ socially-constructed expectations concerning power and the norms of interaction in teaching and learning (Farias et al., 2021). For example, educators’ expectations of independent and proactive learning is deeply embedded in a Socratic teaching philosophy that may not be shared with individuals stemming from other equally valid and culturally-embedded approaches to teaching and learning (Tweed & Lehman, 2002) .

Due to the discipline-specific and dynamic nature of professional interaction, specialist language and communication teaching should be embedded within the discipline, provided at entry to the course and continued throughout a student’s degree (Arkoudis et al., 2012). Particularly for professions-focused degrees, specialised support needs to be easily accessible for students while on placement with a focus on developing advanced sociopragmatic professional language skills.

The educators in this study predominantly identified communication challenges as being linked to highly specific professional communicative situations. This questions the validity of raising the English language course entry requirements or using general academic language proficiency tests (e.g. IELTS) as a graduate measure of professional English language ability. While IELTS and IELTS equivalent tests are reliable for assessing a student’s ability to use the language for functional communication in academic contexts (Dashti & Razmjoo, 2020; Douglas, 2000; Roever, 2011), the findings of this research clearly support Schmitt’s (2005) and O’Loughlin’s (2008) claims that they are not necessarily a reliable indicator of a student’s ability to operate within a professional social work environment.

This study provides a snapshot of placement educator’s experiences of supervising international students on health professions placements. While the study focuses primarily on the context of social work, the findings can easily be extended to other health professions where placement is integral to the degree and a critical aspect of the student’s professional development. Key contributions to the field of international students’ placement education are the development of a systematic means of differentiating between types of communication issues on placement and the identification of sociopragmatic interactional skills as a priority for student learning. Additionally, the analysis provides indications of effective ways for educators to support student’s sociopragmatic confidence, which could form the foundation of professional development programs for educators of international placement students in the health professions. This study does not, however, include the learners’ perspectives of being supervised, particularly in terms of the ways in which their placement educators can best support them in acquiring essential sociopragmatic knowledge of workplace interactional norms. Further research in this area is critical for not only establishing effective teaching methodologies, but also investigating diverse realities of educator roles and educator-student power symmetries. Although some placement educators use direct speech to illustrate conversations they have had with students, the interactions in this study are all retrospectively reported and reliant on the placement educator’s memory of the interaction. Similarly, there is currently no data available to investigate the effectiveness of the dialogic approaches placement educators report to have used. This calls for further research in terms of not only complimenting this data with international student interviews, but also expanding the methodology to collect the actual language used in professional and supervisory interactions.

The sociopragmatic theoretical approach used in this research to further understand the communication issues of international students on placement clearly calls for revisiting a number of current practices in the teaching and learning of international students on placement. The findings reveal a need to question the ubiquitous use of general English language tests (e.g. IELTS) as a predictive or summative measure of professional language ability. Indeed, the focus on general English language testing should be shifted towards developing a symbiotic relationship between placement providers and universities by provision of specialised training for placement educators and specialised sociolinguistic language support for international students while on placement. Not only would such a focus be best suited to addressing communication issues on placement, but it would enhance the likelihood of equitable access to placement opportunities for all students, regardless of their language or cultural backgrounds.
